# PSGL-1: a novel immune checkpoint driving T-cell dysfunction in obstructive sleep apnea

**DOI:** 10.3389/fimmu.2023.1277551

**Published:** 2023-10-03

**Authors:** Elena Díaz-García, Aldara García-Sánchez, Enrique Alfaro, Cristina López-Fernández, Eva Mañas, Irene Cano-Pumarega, Eduardo López-Collazo, Francisco García-Río, Carolina Cubillos-Zapata

**Affiliations:** ^1^ Biomedical Research Networking Centre on Respiratory Diseases (CIBERES), Madrid, Spain; ^2^ Respiratory Diseases Group, Respiratory Diseases Department, Hospital La Paz Institute for Health Research – IdiPAZ, Madrid, Spain; ^3^ Servicio de Neumología, Hospital Universitario Ramón y Cajal, Madrid, Spain; ^4^ The Innate Immune Response Group, Hospital La Paz Institute for Health Research – IdiPAZ, Madrid, Spain; ^5^ Faculty of Medicine, Autonomous University of Madrid, Madrid, Spain

**Keywords:** PSGL-1, T-cells, immunecheckpoints, sleep apnea, intermittent hypoxia, immune surveillance, SIGLEC-5, VISTA

## Abstract

**Introduction:**

Although higher incidence of cancer represents a major burden for obstructive sleep apnea (OSA) patients, the molecular pathways driving this association are not completely understood. Recently, the adhesion receptor P-selectin glycoprotein-1 (PSGL 1) has been identified as a novel immune checkpoint, which are recognized major hallmarks in several types of cancer and have revolutionized cancer therapy.

**Methods:**

The expression of PSGL-1 and its ligands VISTA and SIGLEC-5 was assessed in the leucocytes of OSA patients and control subjects exploring the role of intermittent hypoxia (IH) using *in vitro* models. In addition, PSGL-1 impact on T-cells function was evaluated by *ex vivo* models.

**Results:**

Data showed PSGL-1 expression is upregulated in the T-lymphocytes from patients with severe OSA, indicating a relevant role of hypoxemia mediated by intermittent hypoxia. Besides, results suggest an inhibitory role of PSGL-1 on T-cell proliferation capacity. Finally, the expression of SIGLEC-5 but not VISTA was increased in monocytes from OSA patients, suggesting a regulatory role of intermittent hypoxia.

**Discussion:**

In conclusion, PSGL-1 might constitute an additional immune checkpoint leading to T-cell dysfunction in OSA patients, contributing to the disruption of immune surveillance, which might provide biological plausibility to the higher incidence and aggressiveness of several tumors in these patients.

## 1 Introduction

Obstructive sleep apnea (OSA) is a very prevalent disorder characterized by recurrent episodes of total or partial obstruction of the upper airway during sleep. As consequence, OSA patients exhibit intermittent hypoxemia, increased inspiratory effort and sleep disruption (1–3). Besides, there is growing evidence that OSA is associated with a higher incidence of cancer, tumor aggressiveness and cancer mortality. In fact, the prevalence of cancers in OSA patients reached 1.53 (95%CI, 1.01–2.31) times higher than non-OSA individuals (4–12). In this line, several studies highlight hypoxia as main player in this context. Intermittent hypoxia is widely recognized as a primary contributor to OSA effects on tumor development and progression, enhancing cell proliferation, triggering the release of pro-angiogenic factors, and modifying the immune surveillance system (13–22). The overexpression of hypoxia-inducible factor (HIF-1α) due to intermittent hypoxia (IH) compromises the immune surveillance system by modulating various immune components, which supports the development of a tumor-promoting environment (16, 17). In fact, previous studies carried out in OSA patients demonstrate that HIF-1α triggers the production of transforming growth factor β (TGF-β). Interestingly, this factor plays a crucial role in establishing an immunosuppressive state in the monocytes and natural killer cells of OSA patients (16). Furthermore, the IH induces the expression of the immune checkpoint axis PD-1/PD-L1 (Programmed Cell Death Protein 1/Programmed Cell Death Ligand 1), impairing T-cell function (13, 22). In this line, the potential contribution of additional immune checkpoints to impaired T-cell function in OSA patients remains to be investigated.

In the recent years, immune checkpoints have emerged as major hallmarks of different types of tumors and its inhibition have revolutionized cancer treatment (23). Among the immune checkpoint family, the adhesion receptor P-selectin glycoprotein-1 (PSGL-1) has been recently identified as a key T cell-intrinsic inhibitory receptor (24). Particularly, growing evidence demonstrated that PSGL-1 signaling reduces T cell proliferation (25), activation and survival (26, 27), increases inhibitory receptor expression and dampens T cell receptor (TCR) signals and cytokine production to promote T-cell exhaustion (28). Conversely, PSGL-1 has been typically reported to facilitate adaptive responses by contributing to effector T cell recruitment through selectin binding (29). However, less is known regarding PSGL-1 ligands driving T-cell inhibitory effect (30). In fact, PSGL-1 binds additional molecules other than selectins, including chemokines (CCL19 and CCL21), the sialic acid-binding immunoglobulin-like lectin (SIGLEC)-5 and the V-domain immunoglobulin suppressor of T-cell activation (VISTA) (31–33). The role of selectins and chemokines on PSGL-1 T-cell inhibitory function has been discarded in viral infection models (24), thereby this study focuses on SIGLEC-5 and VISTA. In this context, SIGLEC-5 exerts immune inhibitory roles not only by directly inhibiting T-cell migration by blocking PSGL-1 – selectins binding, but also by reducing T cell receptor TCR-induced activation (34). Moreover, VISTA has been recently identified as a novel promising target in tumor immunotherapy and as a ligand to PSGL-1 under acidic pH-conditions, such as those occurring in the tumor microenvironment.

In this context, we have explored the PSGL-1 pathway activation in OSA patients without clinical evidence of cancer, exploring its contribution to the disruption of immune surveillance prior to tumor initiation. Here, we analyzed PSGL-1 expression in T-cells from patients with OSA and control subjects (CS), addressing its inhibitory roles on T-cell proliferation and activation by *ex vivo* models. Also, we explored the expression of SIGLEC-5 and VISTA as ligands in monocytes from patients with OSA and control subjects and investigated the regulatory role of intermittent hypoxia on the PSGL-1 axis using different *in vitro* models.

## 2 Methods

### 2.1 Study subjects

This study includes 120 OSA patients and 60 control subjects. A detailed description of selection criteria is provided in supplement online. Briefly, OSA diagnosis was determined conducting respiratory polygraphy (using Embletta GOLD, ResMed). This process included continuous monitoring of oronasal airflow, pressure, heart rate, chest and abdominal breathing patterns, and oxygen saturation (SaO_2_). Tests were repeated if patients reported sleeping less than 4 hours or if there was less than 5 hours of nocturnal recording. Patients were categorized as severe OSA when their Apnea-Hypopnea Index (AHI) exceeded 15. Additionally, control subjects (CS), matched for gender and age with OSA patients, were selected from the census register of the Madrid, Spain metropolitan area. Respiratory polygraphy confirmed the absence of OSA in healthy subjects. All participants provided written informed consent and the study was approved by the local ethics committees.

### 2.2 Human cell isolation

Peripheral blood mononuclear cells (PBMCs) were separated through centrifugation, employing a Ficoll-Paque Plus (Amersham Bioscience, Uppsala, Sweden) density gradient. Subsequently, 5×10^6^ PBMCs were seeded into each well of 6-well plates. These cells were then cultured in Roswell Park Memorial Institute (RPMI) 1640 medium supplemented with 100 U/mL penicillin and 100μg/mL streptomycin, along with 10% fetal bovine serum (35). Finally, the cells were incubated for 16 hours at 37°C in a 5% CO_2_ environment.

### 2.3 Flow cytometry

Following a 16-hour incubation, the cells were collected and stained for 30 minutes at 4°C in darkness, using the anti-human antibodies listed in **Table S1**. Subsequently, they were washed with PBS (Phosphate Buffered Saline) containing 1% FBS. Lastly, the cells were acquired using a BD FACS-Celesta flow cytometer (BD-Biosciences, Eysins, Switzerland), and the data were processed with FlowJo vX.0.7 software (FlowJo, USA).

### 2.4 Intermittent hypoxia *in vitro* model

In order to generate the intermittent hypoxia (IH) conditions, we cultured healthy PBMCs in an incubation chamber linked to an external computer-controlled oxygen/nitrogen controller, using the BioSpherix OxyCycler C42 system (Redfield, NY, USA). This system produces cyclical alterations in oxygen levels while maintaining CO_2_ levels, regulating the air gas composition within each chamber (36, 37). In addition, to inhibit Hypoxia Inducible Factor (HIF-1α), two methods were employed: First, monocytes were treated with 30 μM PX-478 (MedKoo Biosciences, Morrisville, NC, USA) for 16 hours (38). Second, cells were transfected using a specific pre-designed silencer for HIF-1α siRNA (s6539, Ambion Inc, Austin, TX, USA) or a control plasmid following the standard protocol for Amaxa™ Human Monocyte Nucleofector Kit (Lonza, Basel, Switzerland). Briefly, cells were transferred to an electroporation cuvette and nucleofected, and cultured for 16h under routine culture conditions or IH conditions. Finally, for the DMOG assay, cells were treated with 100μM of Dimethyloxallyl Glycine (DMOG) for 16 hours in routine culture conditions.

### 2.5 Proliferation *ex vivo* assays

For the proliferation assays 5×10^5^ PBMCs were labeled with Carboxy Fluorescein Succinimidyl Ester (CFSE, ThermoFisher, Darmstadt, Germany) and treated or not with anti-PSGL-1 antibody (Clone KPL-1, Merck Life Science ‘s, Bayswater, Victoria, Australia). The cells were cultured for 4 days and then stained with specific human antibodies for CD4 (APC) or CD8 (APC) (Inmunostep, Salamanca, Spain). Cells were acquired by flow cytometry with the FACS-Calibur flow cytometer (BD-Biosciences, Eysins, Switzerland) and data were analyzed using FlowJo vX.0.7 software (FlowJo, USA).

### 2.6 Statistical analysis

Comparisons between groups were conducted using Mann-Whitney U test, two-way ANOVA with *post-hoc* Tukey’s or Bonferroni tests, or the chi-squared test statistical methods, depending on the nature and distribution of the variables. Correlations were evaluated using Spearman’s rank correlation. To assess data distribution, the Anderson-Darling and D’Agostino-Pearson tests were employed. For all analyses, a significance level of p < 0.05 was applied. Statistical analyses were carried out using Prism 8.0 software (GraphPad, USA) or SPSS (IBM, USA).

## 3 Results

### 3.1 Characteristics of the participants

In this study, 120 patients with OSA were prospectively enrolled and 60 healthy individuals were included as the control group. The key characteristics of the study participants are presented in **Table 1**. Notably, there were no significant differences between the groups in relation to sex, age, BMI, or smoking habits, as detailed in **Table 1**.

**Table 1:** General characteristics of study subject’s.

	Non-apneic healthy subjects (n=60)	Severe OSA patients (n=120)	*p*-value
**Male sex, n (%)**	46 (76.7)	93 (77.5)	0.900
**Age, years**	57 (50-63)	60 (52-69)	0.084
**Body mass index, kg·m^-2^**	32 (26.4-37)	31.67 (28.4-36.2)	0.296
**Smoking habit,n (%)**			0.433
Current smoker	6 (10)	14 (11.7)	
Former smoker	10 (16.7)	14 (11.7)	
Never smoker	44 (73.3)	92 (76.6)	
**Epworth Sleepiness Scale**	2 (0-4)	8.5 (5-12)	<0.001
**AHI, events/h**	3.4 (1.2.6)	50 (38.5-64.1)	<0.001
**ODI, events/h**	3 (0-5)	46.8 (37.7-61.7)	<0.001
**Mean nocturnal SaO_2_, %**	96.1 (94.2-98.4)	90.4 (88.6-91.7)	<0.001
**Low nocturnal SaO_2_, %**	88.3 (81.4-92.5)	75 (65.3-79)	<0.001

Data are expressed as number (percentage) or median (interquartile range). Comparisons between groups were performed by Mann-Whitney U-test or chi-squared test. AHI, apnea-hypopnea index; ODI, oxygen desaturation index; SaO_2_, oxyhemoglobin saturation.

### 3.2 PSGL-1 is overexpressed on T-cells from patients with OSA

**Figure 1:**
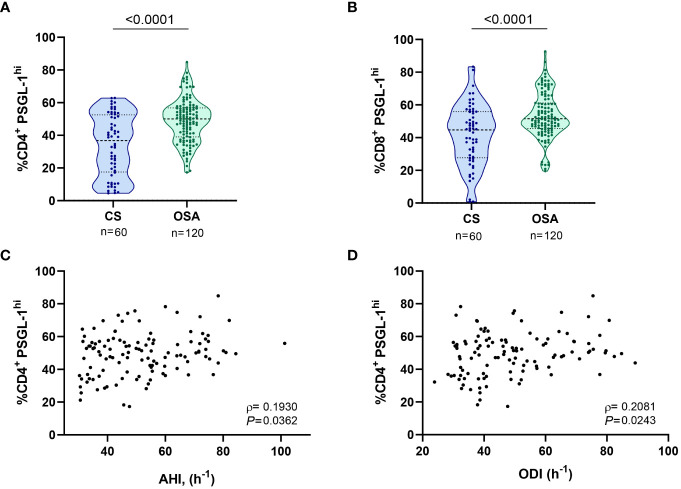
PSGL-1 expression in T-lymphocytes from patients with obstructive sleep apnea. **(A)** Percentage of CD4^+^ T-lymphocytes that expressed high levels of PSGL-1 (PSGL-1^hi^) determined by flow cytometry in control subjects (CS, n=60) and OSA patients (OSA, n=120). **(B)** Percentage of CD8^+^ T-lymphocytes that expressed high levels of PSGL-1 (PSGL-1^hi^) determined by flow cytometry in control subjects (CS, n=60) and OSA patients (OSA, n=120). Comparisons between groups were performed by Mann-Whitney U-test, p-values are shown. **(C, D)** Correlation between the percentage of PSGL-1^hi^ CD4^+^ T-lymphocytes and **(C)** apnea-hypopnea index [AHI] (n=120) and **(D)** oxygen desaturation index [ODI] (n=120). Spearman’s correlation coefficients (ρ) and p-values are shown.

As a first approach, we assessed PSGL-1 expression on T-cells. Our results show that percentage of cells that expressed high levels of PSGL-1 (PSGL-1^hi^, determined as shown in **Figure S1A**) was higher in CD4^+^ and CD8^+^ T-lymphocytes from OSA patients than in those from control subjects (**Figures 1A, B**). Moreover, the percentage of PSGL-1^hi^ CD4^+^ T-cells was related to OSA severity parameters such as the Apnea Hypopnea Index (AHI), Oxygen Desaturation Index (ODI) and mean nocturnal oxygen saturation (mean SatO_2_) (**Figures 1C, D**, **S1B**). Besides, the percentage of PSGL-1^hi^ CD8^+^ T-cells correlated with mean the mean SatO_2_ (**Figure S1C**), although no significant correlation was found with AHI or ODI (data not shown). Finally, PSGL-1 mRNA was overexpressed in PBMCs from OSA patients in comparison with controls subjects and negatively correlated with the mean SatO_2_ (**Figures S1D–E**). Collectively, these data suggested that PSGL-1 expression is elevated in OSA T-cells and related with hypoxia severity.

### 3.3 PSGL-1 expression on T-cells is induced by intermittent hypoxia through HIF-1α

**Figure 2:**
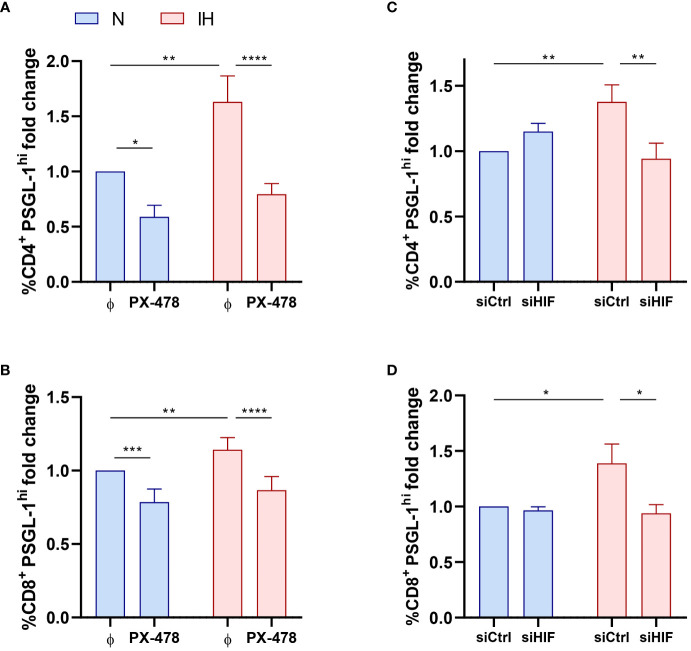
Intermittent hypoxia effect on PSGL-1 expression. **(A, B)** Fold change of the percentage of CD4^+^ **(A)** or CD8^+^ **(B)** T-lymphocytes that expressed high levels of PSGL-1 (PSGL-1^hi^, normalized to the control normoxia condition, CD4^+^: 35.64% ± 5.01%; CD8^+^: 48.40% ± 4.85%) determined by flow cytometry in healthy volunteer’s cells (n=9) treated or not with a specific inhibitor for HIF-1α (PX-478, 30μM) cultured under normoxia (N) or intermittent hypoxia (IH) conditions for 16 hours. **(C, D)** Fold change of the percentage of CD4^+^ **(C)** or CD8^+^ **(D)** T-lymphocytes that expressed high levels of PSGL-1 (PSGL-1^hi^, normalized to the control normoxia condition, CD4^+^: 23.30% ± 2.81%; CD8^+^: 32.51% ± 4.80%) determined by flow cytometry in healthy volunteers’ cells (n=9) transfected with a control silencing RNA (siCtrl) or with a silencing RNA targeting HIF-1α (siHIF), cultured under normoxia (N) or intermittent hypoxia (IH) conditions for 16 hours. Comparisons between groups were performed by Two-way ANOVA with Tukey’s correction for multiple comparison tests. *P<0.05, **P<0.01, ***P<0.001, ****P<0.0001.

Considering that the expression of PSGL-1 is associated to hypoxemia severity, we explore the role of intermittent hypoxia in the upregulation of PSGL-1. As a first approach, we observed that PSGL-1 expression was related to HIF-1α, the master regulator of the molecular response to hypoxia (**Figure S2A**), suggesting an implication of the hypoxia factor in upregulating PSGL-1. Then, we corroborate that OSA patients showed a higher mRNA expression of HIF-1α than control subjects (**Figure S2B**). To further assess this hypoxia role, PBMCs from healthy volunteers were cultured under normoxia or intermittent hypoxia conditions. Concomitantly, in order to clarify the effect of HIF-1α on PSGL-1 expression, we used different approaches targeting HIF-1α expression and/or activity. Firstly, we used the specific agent, PX-478 (S-2-amino-3-[4V-N,N,-bis (2-chloroethyl) amino]-Phenyl Propionic Acid N-oxide Dihydrochloride), which suppresses constitutive and hypoxia-induced levels of HIF-1α (**Figure S2C**). Secondly, HIF-1α expression was silenced using and specific RNA silencer for HIF-1α (siHIF) (**Figure S2D**). Finally, HIF-1α activity was enhanced using DMOG (dimethyloxallyl glycine), a cell permeable prolyl-4-hydroxylase inhibitor that increases endogenous HIF-1α levels as a complementary strategy (**Figure S2E**). Our results showed that exposure to intermittent hypoxia enhanced PSGL-1 expression in both CD4^+^ and CD8^+^ T-cells compared with the cells cultured under normoxic conditions (**Figures 2A–D**). Interestingly, the IH effect was suppressed either when cells were treated with PX-478 or upon HIF-1α silencing, indicating that HIF-1α mediates hypoxia-induced PSGL-1 overexpression in T-cells (**Figures 2A–D**). Indeed, PSGL-1 mRNA expression in healthy PBMCs was also enhanced by IH stimulation and this effect was suppressed when HIF-1α pathway was inhibited (**Figures S2F–G**). Also, PSGL-1 mRNA expression increases when cells are stimulated with DMOG, (**Figure S2H**). Taken together, these data suggest that intermittent hypoxia, through HIF-1α, mediates PSGL-1 overexpression.

### 3.4 Blocking PSGL-1 axis restores T cell proliferation in OSA patients

**Figure 3:**
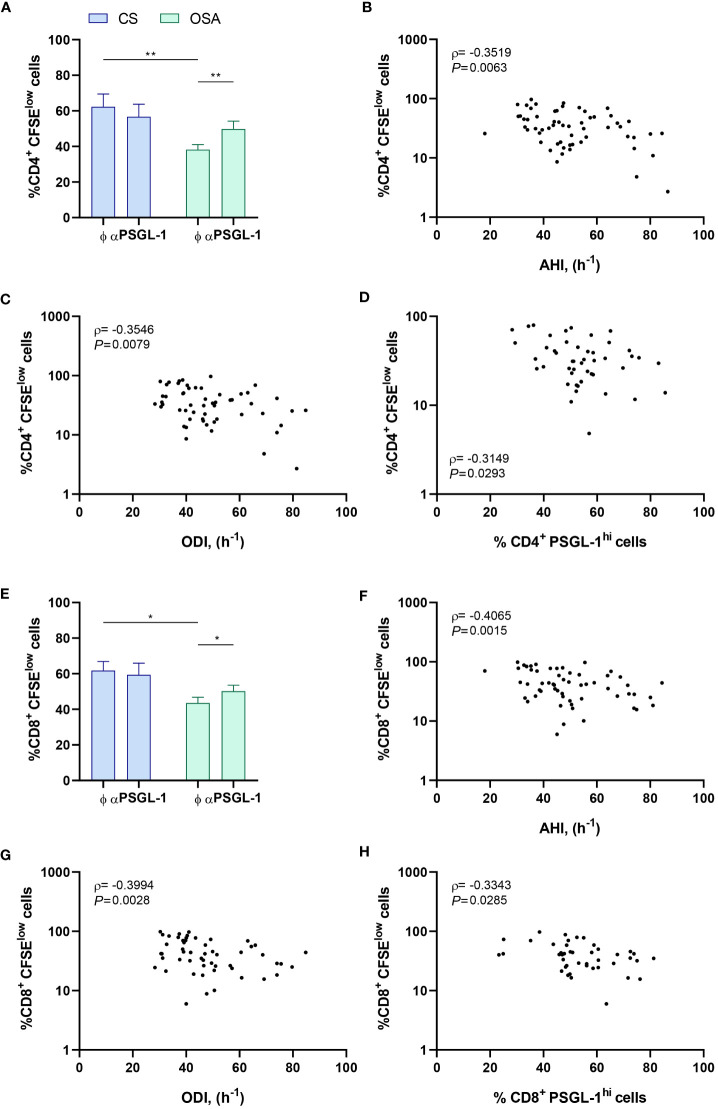
PSGL-1 effect on T-cell proliferation. **(A)** CD4^+^ T-lymphocytes proliferation capacity estimated by flow cytometry using carboxyfluorescein succinimidyl ester (CFSE) staining in cells from control subjects (CS, n=20) or patients with OSA (OSA, n=60) which were cultured for 4 days with or without a neutralizing α-PSGL-1 antibody. **(B–D)** Correlation between the percentage of proliferating (CFSE^low^) CD4^+^ T-lymphocytes and **(B)** apnea-hypopnea index [AHI] (n=60), **(C)** oxygen desaturation index [ODI] (n=60), and **(D)** percentage of CD4^+^ T-lymphocytes that expressed high levels of PSGL-1 (PSGL-1^hi^) determined by flow cytometry. **(E)** CD8^+^ T-lymphocytes proliferation capacity estimated by flow cytometry using CFSE staining in cells from control subjects (CS, n=20) or patients with OSA (OSA, n=60) which were cultured for 4 days with or without a neutralizing α-PSGL-1 antibody. **(F–H)** Correlation between the percentage of proliferating (CFSE^low^) CD8^+^ T-lymphocytes and **(F)** apnea-hypopnea index [AHI] (n=60), **(G)** oxygen desaturation index [ODI] (n=60), and **(H)** percentage of CD8^+^ T-lymphocytes that expressed high levels of PSGL-1 (PSGL-1^hi^) determined by flow cytometry. Comparisons between groups were performed by Two-way ANOVA with Bonferroni’s correction for multiple comparison tests. Spearman’s correlation coefficients (ρ) and p-values are shown. *P<0.05, **P<0.01.

To assess the functional effect of PSGL-1 upregulation on T-cell function, we first evaluated the *ex vivo* proliferation capacity of T-cells from OSA patients and control subjects, blocking or not PSGL-1 axis with a neutralizing αPSGL-1 antibody. In this context, we observed that CD4^+^ T-cell proliferation was significantly impaired in OSA patients in comparison to control subjects (**Figures 3A**, **S3A**). Indeed, the percentage of proliferating T-cells negatively correlated with sleep parameters such as AHI and ODI, suggesting that the proliferation capacity of CD4^+^ T-cell decreases along OSA severity (**Figures 3B, C**). Interestingly, when OSA CD4^+^ T-cells were treated with αPSGL-1 antibody this effect was reverted (**Figure 3A**), indicating that T-cell proliferation impairment in OSA patients may be mediated by PSGL-1. Interestingly, we observed that the percentage of proliferating CD4^+^T-cells is inversely related to the percentage of CD4^+^ T-cells that expressed high levels of PSGL-1 (PSGL-1^hi^) in OSA patients (**Figure 3D**). Besides, CD8^+^ T-cell proliferation was also impaired in OSA patients in comparison to control subjects (**Figures 3E**, **S3B**) and the percentage of proliferating CD8^+^ T-cells negatively correlated with AHI and ODI (**Figures 3F, G**). In addition, CD8^+^ T-cell proliferation was restored after αPSGL-1 antibody treatment (**Figure 3E**) and proliferating CD8^+^T-cells negatively correlated with percentage of PSGL-1^hi^ CD8^+^ T-cells (**Figure 3H**). Altogether, these data indicate that PSGL-1 dampens T-cell proliferation in OSA patients. Moreover, we also found that the percentage of T-cells that expressed high levels of PSGL-1 (PSGL-1^hi^) negatively correlated with plasma levels of immune mediators such as IFN-γ or TNF-α (**Figures S4A–B**). Finally, PSGL-1^hi^ T-cells were related to the exhaustion marker PD-1 (**Figure S4C**), supporting the PSGL-1 role on T-cell function impairment in OSA patients. Altogether these data indicate that PSGL-1 could drive T-cell dysfunction in OSA patients.

### 3.5 Intermittent hypoxia induces T-cell proliferation impairment through PSGL-1 upregulation

**Figure 4:**
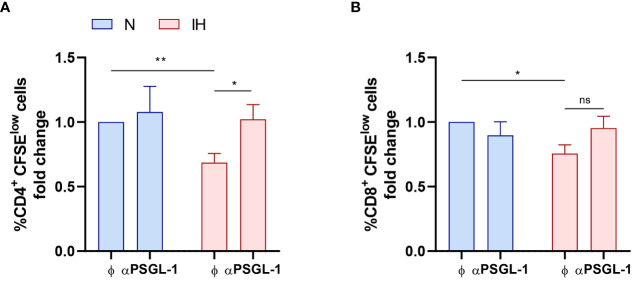
Intermittent hypoxia effect on T-cell proliferation. Fold change of **(A)** CD4^+^ and **(B)** CD8^+^ T-lymphocytes proliferation capacity (normalized to the control normoxia condition, CD4^+^: 47.13% ± 7.66%; CD8^+^: 64.46% ± 8.48%) estimated by flow cytometry using carboxyfluorescein succinimidyl ester (CFSE) staining in cells from healthy volunteers (n=9) which were cultured for 4 days with or without a neutralizing α-PSGL-1 antibody under normoxia (N) or intermittent hypoxia conditions (IH). Comparisons between groups were performed by Two-way ANOVA with Tukey’s correction for multiple comparison tests.*P<0.05, **P<0.01, ns, non-significant.

To further check whether T-cell proliferation impairment was mediated by intermittent hypoxia and subsequent PSGL-1 overexpression; we performed an *in vitro* proliferation assay using T-cells from healthy volunteers. Strikingly, exposure to intermittent hypoxia dampens the proliferation of healthy CD4^+^ T-cells and PSGL-1 axis blockade suppressed this effect (**Figure 4A**). Moreover, IH also decreases CD8^+^ T-cell proliferation. However, although PSGL-1 axis blockade induce a tendency to proliferation recovery, it did not reach statistical significance (**Figure 4B**). Collectively, these results suggest that IH, through PSGL-1 overexpression contribute to dampen T-cell proliferation in OSA patients.

### 3.6 SIGLEC-5 is overexpressed on monocytes from patients with OSA

**Figure 5:**
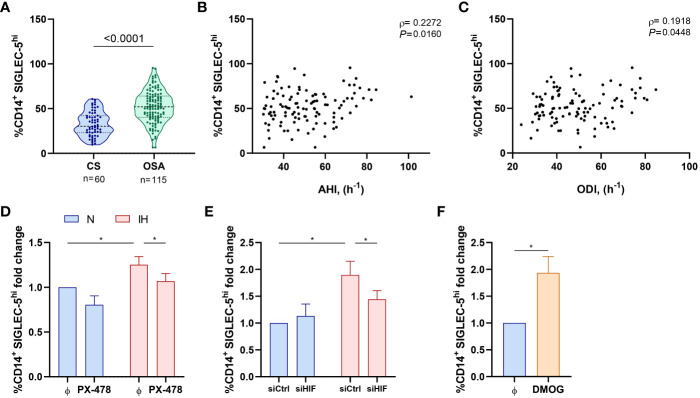
SIGLEC-5 is overexpressed in patients with obstructive sleep apnea. **(A)** Percentage of monocytes (CD14^+^) that expressed high levels of SIGLEC-5 (SIGLEC-5^hi^) determined by flow cytometry in control subjects (CS, n=60) and OSA patients (OSA, n=115). Comparison between groups was performed by Mann-Whitney U-test, p-value is shown. **(B, C)** Correlation between the percentage of SIGLEC-5^hi^ monocytes and **(B)** apnea-hypopnea index [AHI] (n=115) and **(C)** oxygen desaturation index [ODI] (n=115). Spearman’s correlation coefficients (ρ) and p-values are shown. **(D)** Fold change of the percentage of monocytes that expressed high levels of SIGLEC-5 (SIGLEC-5^hi^, normalized to the control normoxia condition, 41.93% ± 7.92%) determined by flow cytometry in healthy volunteer’s cells (n=11) treated or not with a specific inhibitor for HIF-1α (PX-478, 30μM) cultured under normoxia (N) or intermittent hypoxia (IH) conditions for 16 hours. **(E)** Fold change of the percentage of monocytes that expressed high levels of SIGLEC-5 (SIGLEC-5^hi^, normalized to the control normoxia condition, 32.38% ± 4.35%) determined by flow cytometry in healthy volunteers’ cells (n=7) transfected with a control silencing RNA (siCtrl) or with a silencing RNA targeting HIF-1α (siHIF), cultured under normoxia (N) or intermittent hypoxia (IH) conditions for 16 hours. Comparisons between groups were performed by Two-way ANOVA with Tukey’s correction for multiple comparison tests. **(F)** Fold change of the percentage of monocytes that expressed high levels of SIGLEC-5 (SIGLEC-5^hi^, normalized to the control normoxia condition, 41.93% ± 7.92%) determined by flow cytometry in healthy volunteers’ cells treated or not with 100μM of dimethyloxallyl glycine (DMOG) for 16 hours in routine culture conditions (n=7) *P<0.05.

We then assessed the expression on monocytes of SIGLEC-5 and VISTA as potential PSGL-1 ligands driving T-cell impairment. Our data indicate an overexpression of SIGLEC-5 but not VISTA in monocytes from OSA patients in comparison to control subjects (**Figures 5A**, **S5A, B**). In this line, the expression of VISTA did not correlate with OSA severity parameters (data not shown), while the percentage of monocytes that expressed high levels of SIGLEC-5 (SIGLEC-5^hi,^determined as shown in **Supplementary Figure 5A**) positively correlated to AHI and ODI parameters, indicating that SIGLEC-5 increases along OSA severity (**Figures 5B, C**). Accordingly, SIGLEC-5 mRNA expression was higher in monocytes from OSA patients and negatively correlated with the mean SatO_2_ (**Figures S5C–D**). Collectively, these data suggested that SIGLEC-5 expression is elevated in OSA monocytes and related with hypoxia severity.

### 3.7 SIGLEC-5 overexpression is induced by intermittent hypoxia

Given that SIGLEC-5 is upregulated in OSA monocytes, we then explored intermittent hypoxia role in this context. Interestingly, we found that SIGLEC-5 expression was related to HIF-1α mRNA expression (**Figure S5E**). In this context, we explored the potential role of hypoxemia in the SIGLEC-5 upregulation, using the intermittent hypoxia *in vitro* model in combination with the HIF-1α inhibitor PX-478 or the specific RNA silencer (siRNA) for HIF-1α (siHIF). Our data indicate that monocytes cultured under intermittent hypoxia exhibit higher levels of SIGLEC-5 when compared to cells cultured under normoxia condition (**Figures 5D, E**). Interestingly, this effect was blocked when cells were treated with PX-478 or transfected with the HIF-1α siRNA (**Figures 5D, E**). Moreover, this effect was also observed at the mRNA level (**Figures S5F–G**). Finally, DMOG treatment also enhanced SIGLEC-5 expression both at the protein and mRNA level (**Figures 5F**, **S5H**). Collectively, these results suggest that intermittent hypoxia, through HIF-1α, could mediate SIGLEC-5 upregulation in OSA monocytes.

## 4 Discussion

The higher risk of cancer represents a major burden for sleep apnea patients. In this study, we have explored the PSGL-1 expression as an immune checkpoint and its capacity to reduce T-cell function. In addition, we analyzed the intermittent hypoxia effect on the PSGL-1 expression and function using hypoxemia clinical parameters and *ex vivo* and *in vitro* strategies. Ultimately, in order to have a complete picture of the PSGL-1 function, we analyzed known potential ligands in monocytes, however, only SIGLEC-5 was overexpressed in OSA monocytes and associated with hypoxemia conditions. Overall, this study suggests that OSA patients exhibited an impaired T cell response through PSGL-1, providing a reasonable explanation to understand the impairment of immune surveillance in these patients.

### 4.1 Impact of the intermittent hypoxia in PSGL-1 pathway

The hypoxemia is one of the main intermediate mechanisms of OSA. In this regard, a recent study reported that PSGL-1 expression increased susceptibility in patients with acute respiratory distress syndrome, demonstrating the PSGL-1 promoter activity was strongly regulated by HIF-1α and HIF-2α (39). Remarkably, hypoxia develops in most solid tumors because of the rapid growth of the tumor that outstrips the oxygen supply, and is one of the main hallmarks of tumor microenvironment (40). In this line, we speculate that the effect of hypoxia may directly or indirectly impact tumor growth in OSA patients. Nevertheless, additional studies are needed to understand the implications of PSGL-1 axis in the context of OSA-related tumor initiation. On the other side, Sun et al., suggest that additional inflammatory and epigenetic factors may regulate PSGL-1 expression (39). Moreover, previous studies indicate an upregulation of PSGL-1 in acute inflammation (41). In line, we previously demonstrated a systemic inflammation in OSA patients resulting from the inflammasome activation (42, 43), also, others authors reported the low-grade-chronic inflammatory state in these patients (44–49). On the other hand, there are two studies in OSA patients focusing on potential role of circulating PSGL-1, as a driver of leucocyte infiltration facilitating the development of atherosclerosis; however, assessing PSGL-1 circulating levels provided discrepant results (50, 51). Particularly, Horvath et al., reported no differences on circulating PSGL-1 levels, while Winiarska et al., concluded that circulating PSGL-1 levels were significantly increased in OSA patients and correlated with AHI. In this line, our study provides new insight by assessing PSGL-1 expression on leucocyte membrane, where it exerts its biological function (28). More importantly, the present study focused on recently described PSGL-1 immune checkpoint function, addressing its potential contribution as a negative regulator of T-cell function in OSA patients, possibly eliciting cancer development and progression.

### 4.2 Connections to T-cell dysfunction

Previous studies performed in animal models have shown that genetic deletion of PSGL-1 enhanced CD4^+^ and CD8^+^ T-cell responses by preventing development of exhausted T-cells, while increasing T-cell effector function and decreasing inhibitory receptor expression (24, 25, 52, 53). Moreover, many efforts have focused in unravelling the signaling pathway driving T-cell exhaustion upon PSGL-1 engagement. So far, PSGL-1 ligation on exhausted T-cells resulted in diminished ERK (extracellular signal-regulated kinases) and AKT (Protein kinase B) signaling (24), and constrains its metabolic activity, thereby, limiting anti-tumor response (54). In addition, PSGL-1 has been shown to suppress the expression of TCF1 (T cell factor 1), a transcription factor with a key role in the self-renewal, expansion, and development of effector function in T-cells. Simultaneously, PSGL-1 also enhances the expression of TOX (thymocyte selection-associated high mobility group box factor), a pivotal controller of T-cell irreversible exhaustion (54). Furthermore, PSGL-1 has been reported to act upstream of PD-1 (another immune checkpoint), requiring direct interaction with TCR to directly restrain its signaling, thus attenuating T-cell activation (54). Interestingly, PD-1/PD-L1 pathway has also been shown to be implicated in T-cell decreased proliferation in OSA subjects (13). In agreement, our results showed that although the blockage of PSGL-1 pathway significantly recovers T-cell function, is a partial recovery not reaching the control subject proliferation levels. This indicates that other pathways, such as PD-1/PD-L1 may be implicated in T-cell dysfunction in OSA patients. In agreement, recent study showed that PD-1 immune checkpoint blockade and PSGL-1 inhibition synergize to reinvigorate exhausted T cells (55). Indeed, in animal models PSGL-1 blockade reduced anti-PD-1 resistant melanoma tumor growth, one of the most common types of tumor among OSA patients (12, 54, 56). Altogether, this evidence suggests that the impairment of T- lymphocyte function in OSA patients involve the activity of both immune checkpoints, PSGL-1 and PD-1/PD-L1.

### 4.3 PSGL-1 ligands: VISTA

In this context, a key outstanding question is the ligand driving PSGL-1 mediated T-cell dysfunction. Therefore, we have focused on PSGL-1 ligand candidates SIGLEC-5 and VISTA, as previous studies have reported (32, 33). Interestingly, VISTA is a well-established immune regulatory receptor independently of PSGL-1 (57–59). Indeed, VISTA promotes the suppressive function of myeloid-derived suppressor cells in the tumor microenvironment dampening T-cell response (60). In this line, the role of VISTA on myeloid cells is complex and remains to be completely understood. For instance, while overexpression of VISTA on monocytes induced elevated levels of cytokine expression (61), VISTA deficient myeloid cells showed an enhanced inflammatory phenotype (62). Also, VISTA-deficient myeloid cells showed a marked dysregulation in the surface expression of chemokine receptors, and their responses to inflammatory chemokines is profoundly impaired. Altogether this evidence underscores a central function of VISTA controlling both innate and adaptive immunity (63). Besides, Deng et al., reported that VISTA is preferentially upregulated under hypoxic conditions, through direct binding of HIF-1α to VISTA gene promoter (60), which prompted us to speculate that this molecule could play a central role engaging PSGL-1 in OSA patients. Intriguingly, we did not find an upregulation of VISTA in OSA monocytes. In this line, a previous study reported a downregulation of VISTA in the microglia from patients with sepsis and in chronic multiple sclerosis lesions (64). Moreover, evidence showed that VISTA was downregulated under certain inflammatory conditions (e.g., stimulation by LPS, CFA, and poly-IC) (57, 64). Given that OSA patients exhibit a proinflammatory state, it is plausible that inflammation and hypoxia are counteracting each other, resulting in no differences in VISTA expression. Besides, current studies have shown that PSGL-1-VISTA interaction occurs in a pH-dependent manner (pH < 6.2) *in vitro*, so that binding *in vivo* has yet to be confirmed. Moreover, VISTA seems to utilize pH sensitivity to suppress T-cell function primarily in acidic and inflamed environments like tumors, rather than in lymphoid organs or the bloodstream (33). In this line, the OSA patients included in this study had no evidence of cancer at the time of recruitment, also, VISTA analysis was performed in circulating monocytes where the pH do not reach the acidic conditions as happen in tumor microenvironment (TME), so, we speculate that probably VISTA could not engage PSGL-1 under this conditions. Nevertheless, VISTA may probably function as an additional ligand for PSGL-1 under acidic TME conditions, enhancing PSGL-1 overexpression effect in OSA patients with stablished tumors. However, further evidence is needed to uncover the precise role of VISTA in this context.

### 4.4 PSGL-1 ligands: SIGLEC-5

Furthermore, SIGLEC-5 is upregulated in several types of tumors, including glioma and colorectal cancer and has been proposed as a prognosis marker to predict patient outcome (65, 66). Indeed, SIGLEC-5 has been reported to exert an anti-inflammatory role by directly blocking PSGL-1 interaction with selectins, hindering leukocyte infiltration (32). Additionally, SIGLEC-5 suppresses T cell activation by abrogating antigen receptor induced activation of NFAT (nuclear factor of activated T cells) and AP-1 (activating protein-1), independently of PSGL-1 (34). In turn, SIGLEC-5 also suppresses the inflammatory response of innate immune cells, such as monocytes (67–69). As a consequence, the interaction between PSGL-1 and SIGLEC-5 may not only impair T-cell function but also impact the innate subset, inducing a broad immunosuppressive state, thus facilitating tumor progression. Moreover, it has been shown that Siglec-expressing cells are specifically recruited to the TME (70), were they could fulfill specific functions in the tumor progression (71, 72). Herein, we showed that SIGLEC-5 is upregulated in OSA monocytes, also, its expression significantly increased along the hypoxemia severity. Indeed, we corroborated by *in vitro* assays the relevant role of the hypoxemia in this context. In agreement, previous evidence suggested that blocking SIGLEC-5 could serve as a new immune checkpoint blockade strategy to enhance anti-tumor T cell functions (34). Thus, we have performed a SIGLEC-5 *ex vivo* assay to explore the antigen presentation role; however, our data not show a significant effect on the lymphocyte proliferation using a functional blocking anti-SIGLEC-5 (data not shown). Furthermore, there is scarce literature assessing SIGLEC-5 interaction with PSGL-1 and its role as an immune checkpoint; probably because SIGLEC-5 is not present in mice, and much of what is known regarding PSGL-1 role in T-cell dysfunction is based on mouse models rather. Therefore, further investigations are needed either to understand SIGLEC-5 and VISTA PSGL-1 engaging; or to unravel other yet-to-be-identified ligands, which could potentially contribute to PSGL-1 mediated T-cell exhaustion.

### 4.5 Limitations

Our study has several limitations, which we acknowledge. First, the diagnosis of OSA in patients was based on validated respiratory polygraphy, and while we believe their clinical characterization is sufficient, this method does not enable us to evaluate the role of sleep fragmentation in the upregulation of PSGL-1. Second, while our data demonstrate that IH modulate PSGL-1 and SIGLEC-5 expression, it is predictable that other pathways contribute to modulate PSGL-1 expression. Third, although our study demonstrates an upregulation of SIGLEC-5 it does not provide information about its potential engaging PSGL-1 and/or modulating T-cell function. Fourth, on our *in vitro* model the intermittent hypoxia cycles are longer than those suffered by OSA patients. Fifth, our study includes patients without any evidence of cancer, so we cannot conclude PSGL-1 effect on tumor development or progression. Sixth, this study does not provide any information on the effect of OSA treatment on PSGL-1 expression or its effect on the development or progression of cancer.

### 4.6 Conclusions

**Figure 6:**
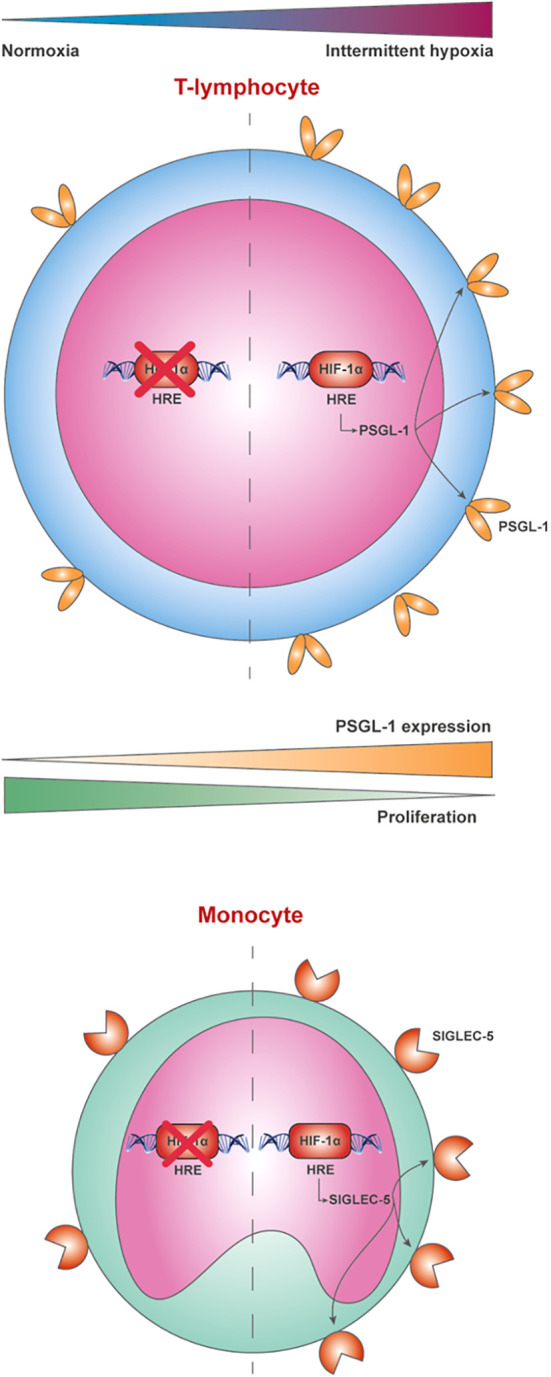
P-selectin glycoprotein-1 (PSGL-1) is overexpressed in lymphocytes from patients with severe OSA through a hypoxemia-dependent mechanism mediated by HIF-1α. The behavior of PSGL-1 as an immune checkpoint leading to T-cell dysfunction appears to be mediated through the expression of the sialic acid-binding immunoglobulin-like lectin (SIGLEC-5) in monocytes, which reduces T-cell proliferation, compromising the immunosurveillance system of these patients.

In conclusion, this study demonstrates that PSGL-1 expression is upregulated in the lymphocytes from patients with severe OSA, indicating a relevant role of the hypoxemia by HIF-1α mediation. Furthermore, our data showed that PSGL-1 could constitute an additional immune checkpoint leading to T-cell dysfunction in OSA patients, thereby potentially contributing to the higher cancer incidence and aggressiveness. Finally, our data show an upregulation of SIGLEC-5 in OSA monocytes, suggesting it potential contribution to the PSGL-1 axis and highlighting the need of further studies assessing PSGL-1 engaging (**Figure 6**).

## Data availability statement

The raw data supporting the conclusions of this article will be made available by the authors, without undue reservation.

## Ethics statement

The studies involving humans were approved by CEIm Hospital Universitario La Paz (PI-3620). The studies were conducted in accordance with the local legislation and institutional requirements. The participants provided their written informed consent to participate in this study.

## Author contributions

ED-G: Data curation, Formal Analysis, Investigation, Methodology, Supervision, Writing – original draft, Writing – review & editing. AG-S: Conceptualization, Data curation, Investigation, Methodology, Formal Analysis, Software, Writing – original draft, Writing – review & editing. EA: Data curation, Formal Analysis, Methodology, Writing – review & editing. CL-F: Methodology, Writing – review & editing. EM: Formal Analysis, Methodology, Writing – review & editing. IC-P: Conceptualization, Formal Analysis, Methodology, Writing – review & editing. EL-C: Conceptualization, Resources, Validation, Writing – review & editing. FG-R: Conceptualization, Formal Analysis, Funding acquisition, Investigation, Project administration, Resources, Supervision, Validation, Writing – original draft, Writing – review & editing. CC-Z: Conceptualization, Data curation, Formal Analysis, Funding acquisition, Investigation, Methodology, Project administration, Resources, Supervision, Validation, Writing – original draft, Writing – review & editing.

## Glossary

**Table:**

OSA	Obstructive sleep apnea
HIF-1α	hypoxia-inducible factor-1 alpha
IH	intermittent hypoxia
TGF-β	transforming growth factor β
PD-1/PD-L1	programmed Cell Death Protein 1/programmed Cell Death Ligand 1
PSGL-1	P-selectin glycoprotein-1
TCR	T cell receptor
CCL19	Chemokine (C-C motif) ligand 19
SIGLEC-5	sialic acid- binding immunoglobulin-like lectin 5
VISTA	V-domain immunoglobulin suppressor of T- cell activation
CS	controls subjects
AHI	apnea-hypopnea index
ODI	oxygen desaturation index
SaO_2_	oxygen saturation
PBMCs	peripheral blood mononuclear cells
CFSE	carboxyfluorescein succinimidyl ester
PX-478	S-2-amino-3-[4V-N,N,-bis(2-chloroethyl) amino]-phenyl propionic acid N-oxide dihydrochloride
DMOG	dimethyloxallyl glycine
IFN	interferon
TNF	tumor necrosis factor
LPS	lipopolysaccharide
ERK	extracellular signal-regulated kinases
AKT	protein kinase B
TCF1	T cell factor 1
TOX	thymocyte selection-associated high mobility group box factor
CFA	complete Freund’s adjuvant
poly-IC	Polyinosinic-polycytidylic acid
TME	tumor microenvironment
NFAT	nuclear factor of activated T cells
AP-1	activating protein-1.
