# Supplementary material for: PSGL-1: a novel immune checkpoint driving T-cell dysfunction in obstructive sleep apnea
Source: Front Immunol. 2023 Oct 3;14:1277551. doi: 10.3389/fimmu.2023.1277551 (PMC10579800; doi:10.3389/fimmu.2023.1277551)
Supplement: Supplementary file 1 [file DataSheet_1.docx]

Supplementary Material

# Supplementary Data

## Study subjects

# 120 recently diagnosed severe OSA patients were consecutively recruited from the Pneumology Service of La Paz-Cantoblanco University Hospital and Ramón y Cajal University Hospital, Madrid, Spain. Patients aged between 40 and 65 years with an apnea-hypopnea index (AHI) > 30 events/h were included in the study. Exclusion criteria were the following: previous or current treatment with oxygen or mechanical ventilation; underweight patients (body mass index [BMI] <18.5 Kg/m2) or those with morbid obesity (BMI >40 Kg/m^2^); history of respiratory disease, including chronic obstructive pulmonary disease, asthma or respiratory failure; any infectious disease in the previous 3 months; and the use of inhaled or systemic corticosteroids or other anti-inflammatory drugs. The subjects were classified as current smokers (defined as daily smoking of any number of cigarettes), former smokers (defined as patients who stopped smoking at least 6 months before study inclusion), and nonsmokers. As a control group, healthy volunteers were selected who were homogeneous in sex, age, smoking habit and BMI. None of these volunteers were being treated with any type of medication, and the diagnosis of OSA was ruled out by respiratory polygraphy. The study was approved by the Hospital Universitario La Paz Ethics Committee (PI-3643) and extended to Ramón y Cajal University Hospital.

## mRNA isolation and quantification by qPCR

# RNA was obtained from PBMCs using the High Pure RNA Isolation Kit (Roche Diagnostics, Switzerland). In both cohorts, RNA was quantified and complementary DNA (cDNA) was obtained by reverse transcription of 1ug RNA using the High-Capacity cDNA Reverse Transcription kit (Applied Biosystems, Waltham, USA). cDNA levels were measured using CFX96 Touch Real-Time PCR Detection System (Bio-Rad Laboratories, Hercules, CA, USA), NZYSupreme qPCR Green Master Mix (2x) (NZYTech, Portugal) and specific primers indicated in supplementary Table 2. The results were normalized to the expression of 18S, and the cDNA copy number of each gene of interest was determined using a 6-point standard curve.

## Plasma Cytokine Determination

# The blood samples were centrifuged to separate plasma, and all specimens were immediately aliquoted, frozen and stored at -80°C. IFN-γ and TNF-α plasma concentration was quantified by cytometry bead array technology using commercial kits (558279 and 558269, BD-Biosciences, Eysins, Switzerland), following the manufacturer’s protocol. The samples were collected by flow cytometry using a BD FACS Calibur flow cytometer (BD-Biosciences, Eysins, Switzerland) and analyzed FCAP array (BD-Biosciences, Eysins, Switzerland).

# Supplementary Figures and Tables

## Supplementary Tables

### Supplementary Table 1. Flow cytometry anti-human antibodies used in the study

| Target | Fluorochrome | Manufacturer | Reference |
| --- | --- | --- | --- |
| CD4 | PerCP-Cy^TM^5.5 | BD-Biosciences  Eysins, Switzerland | 566923 |
| CD8 | BV711 |  | 563667 |
| CD14 | BV510 |  | 563079 |
| PSGL-1 | BUV737 |  | 748330 |
| SIGLEC-5 | BV650 |  | 749695 |
| VISTA | BV421 |  | 566751 |
| PD-1 | BV785 | Biolegend (San Diego, CA, USA) | 329929 |

### Supplementary Table 2. qPCR Primer sequences used in the study

| 18S | Forward primer | CGGCGACGACCCATTCGAAC |
| --- | --- | --- |
|  | Reverse primer | GAATCGAACCCTGATTCCCCGTC |
| PSGL-1 | Forward primer | CATGGAGTTCGTGGTGACCC |
|  | Reverse primer | CATGGCACCAGCCATCTTATCTC |
| SIGLEC-5 | Forward primer | CAAGGGAGATCGAACCTCGG |
|  | Reverse primer | TGCGGGCTTTCACTATTAAAAAGA |
| HIF-1α | Forward primer | TTCCAGTTACGTTCCTTCGATCA |
|  | Reverse primer | TTTGAGGACTTGCGCTTTCA |

## Supplementary Figures

##

# Supplementary Figure 1. (A) Gating strategy for cytometry determination of the percentage of CD4^+^ and CD8^+^ T-lymphocytes that expressed high levels of PSGL-1 (PSGL-1^hi^). Blue plots corresponding to CS subjects. Green plots corresponding to OSA subjects. (B) Correlation between the percentage of PSGL-1^hi^ CD4^+^ T-lymphocytes and mean nocturnal oxyhemoglobin saturation [mean SaO_2_] (n=120). (C) Correlation between the percentage of PSGL-1^hi^ CD8^+^ T-lymphocytes from OSA patients and mean nocturnal oxyhemoglobin saturation [mean SaO_2_] (n=120). Spearman’s correlation coefficients (ρ) and p-values are shown. (D) PSGL-1 mRNA levels in PBMCs from control subjects (CS, n=50) and OSA patients (OSA, n=100) determined by qPCR. Comparison between groups was performed by Mann-Whitney U-test, p-value is shown. (E) Correlation between PSGL-1 mRNA levels in PBMCs from OSA patients and mean nocturnal oxyhemoglobin saturation [mean SaO_2_] (n=100). Spearman’s correlation coefficient (ρ) and p-value is shown.

#

# Supplementary Figure 2. (A) Correlation between PSGL-1 and HIF-1α mRNA levels in PBMCs from OSA patients (n=100). Spearman’s correlation coefficients (ρ) and p-values are shown. (B) HIF-1α mRNA levels in PBMCs from control subjects (CS, n=25) and OSA patients (OSA, n=100) determined by qPCR. Comparison between groups was performed by Mann-Whitney U-test, p-value is shown. (C) Fold change of mRNA HIF-1α expression normalized to the normoxia-control condition (16.41±3.87) determined by qPCR in healthy volunteer’s PBMCs (n=6) treated or not with a specific inhibitor for HIF-1α (PX-478, 30μM) cultured under normoxia (N) or intermittent hypoxia (IH) conditions for 16 hours. (D) Fold change of mRNA HIF-1α expression normalized to the normoxia-control condition (26.96±6.94) determined by qPCR in healthy volunteer’s PBMCs (n=6) transfected with a control silencing RNA (siCtrl) or with a silencing RNA targeting HIF-1α, cultured under normoxia (N) or intermittent hypoxia (IH) conditions for 16 hours. (E) Fold change of mRNA VEGF (vascular endothelial growth factor) expression normalized to the normoxia-control condition (2.93±1.45) determined by qPCR in healthy volunteer’s PBMCs (n=6) treated or not with dimethyloxallyl glycine (DMOG) for 16 hours. (F) Fold change of mRNA PSGL-1 expression normalized to the normoxia-control condition (0.15±0.08) determined by qPCR in healthy volunteer’s PBMCs (n=6) treated or not with a specific inhibitor for HIF-1α (PX-478, 30μM) cultured under normoxia (N) or intermittent hypoxia (IH) conditions for 16 hours. (G) Fold change of mRNA PSGL-1 expression normalized to the normoxia-control condition (0.44±0.11) determined by qPCR in healthy volunteer’s PBMCs (n=6) transfected with a control silencing RNA (siCtrl) or with a silencing RNA targeting HIF-1α, cultured under normoxia (N) or intermittent hypoxia (IH) conditions for 16 hours. (H) Fold change of mRNA PSGL-1 expression normalized to the normoxia-control condition (0.15±0.08) determined by qPCR in healthy volunteer’s PBMCs (n=6) treated or not with dimethyloxallyl glycine (DMOG) for 16 hours. Comparisons between groups were performed by Paired T-test or Two-way ANOVA with Tukey’s correction for multiple comparison tests. *: P<0.05, **: P<0.01

#

# Supplementary Figure 3. Gating strategy for cytometry determination of the percentage of proliferating CD4^+^ (A) and CD8^+^ (B) T-lymphocytes expressing low levels of carboxyfluorescein succinimidyl ester (CFSE^low^). Blue plots corresponding to CS subjects. Green plots corresponding to OSA subjects.

#

# Supplementary Figure 4. (A) Correlation between plasma levels interferon gamma (IFN-γ) determined by cytometric bead array (CBA) and the percentage of CD4^+^ (upper panel) and CD8^+^ (lower panel) T-lymphocytes that expressed high levels of PSGL-1 (PSGL-1^hi^) determined by flow cytometry in OSA patients (n=80). (B) Correlation between plasma levels tumor necrosis factor alpha (TNF-α) determined by CBA and the percentage of CD4^+^ (upper panel) and CD8^+^ (lower panel) T-lymphocytes that expressed high levels of PSGL-1 (PSGL-1^hi^) determined by flow cytometry in OSA patients (n=80). (C) Correlation between the percentage of CD4^+^ (upper panel) and CD8^+^ (lower panel) T-lymphocytes that expressed PD-1 (PD-1^+^) and the percentage of CD4^+^ and CD8^+^ T-lymphocytes, respectively, that expressed high levels of PSGL-1 (PSGL-1^hi^) determined by flow cytometry in OSA patients (n=80). Spearman’s correlation coefficients (ρ) and p-values are shown.

#

# Supplementary Figure 5. (A) Gating strategy for cytometry determination of the Percentage of monocytes (CD14^+^) that expressed high levels of SIGLEC-5 (SIGLEC-5^hi^) determined by flow cytometry in control subjects (CS, n=60) and OSA patients. Blue plot corresponding to CS subject. Green plot corresponding to OSA subject. (B) Percentage of monocytes (CD14^+^) that expressed high levels of VISTA (VISTA^hi^) determined by flow cytometry in control subjects (CS, n=60) and OSA patients (n=115). (C) SIGLEC-5 mRNA levels in PBMCs from control subjects (CS, n=55) and OSA patients (OSA, n=110) determined by qPCR. Comparisons between groups were performed by Mann-Whitney U-test, p-values are shown. (D) Correlation between SIGLEC-5 mRNA levels in PBMCs from OSA patients and mean nocturnal oxyhemoglobin saturation [mean SaO_2_] (n=110). (E) Correlation between SIGLEC-5 and HIF-1α mRNA levels in PBMCs from OSA patients (n=100). Spearman’s correlation coefficients (ρ) and p-values are shown. (F) Fold change of mRNA SIGLEC-5 expression normalized to the normoxia-control condition (0.57±0.30) determined by qPCR in healthy volunteer’s monocytes (n=4) treated or not with a specific inhibitor for HIF-1α (PX-478, 30μM) cultured under normoxia (N) or intermittent hypoxia (IH) conditions for 16 hours. (G) Fold change of mRNA SIGLEC-5 expression normalized to the normoxia-control condition (0.81±0.28) determined by qPCR in healthy volunteer’s monocytes (n=6) transfected with a control silencing RNA (siCtrl) or with a silencing RNA targeting HIF-1α, cultured under normoxia (N) or intermittent hypoxia (IH) conditions for 16 hours. (H) Fold change of mRNA SIGLEC-5 expression normalized to the normoxia-control condition (0.57±0.30) determined by qPCR in healthy volunteer’s PBMCs (n=6) treated or not with dimethyloxallyl glycine (DMOG) for 16 hours. Comparisons between groups were performed by Paired T-test or Two-way ANOVA with Tukey’s correction for multiple comparison tests. *: P<0.05, **: P<0.01.
